# Multiplex polymerase chain reaction to diagnose bloodstream infections in patients after cardiothoracic surgery

**DOI:** 10.1186/s12871-019-0727-5

**Published:** 2019-04-23

**Authors:** Kevin Pilarczyk, Peter-Michael Rath, Joerg Steinmann, Matthias Thielmann, Stephan A. Padosch, Max Dürbeck, Heinz Jakob, Fabian Dusse

**Affiliations:** 1 Department of Intensive Care Medicine, imland Klinik Rendsburg managed by Sana GmbH, Rendsburg, Germany; 2 Department of Anaesthesiology and Intensive Care Medicine, University Hospital of Cologne, Kerpener Str. 62, 50937 Köln, Germany; 3 Department of Thoracic and Cardiovascular Surgery, West German Heart and Vascular Center Essen, University Hospital Essen, University of Duisburg-Essen, Essen, Germany; 4 Institute of Medical Microbiology, University Hospital Essen, University of Duisburg-Essen, Essen, Germany; 5 Institute of Clinical Hygiene, Medical Microbiology and Infectiology, Paracelsus Medical University, Nuremberg, Germany

**Keywords:** Blood stream infection, Blood culture, Real time multiplex polymerase chain reaction

## Abstract

**Background:**

Sepsis and other infectious complications are major causes of mortality and morbidity in patients after cardiac surgery. Whereas conventional blood culture (BC) suffers from low sensitivity as well as a reporting delay of approximately 48–72 h, real-time multiplex polymerase chain reaction (PCR) based technologies like “SeptiFast” (SF) might offer a fast and reliable alternative for detection of bloodstream infections (BSI). The aim of this study was to compare the performance of SF with BC testing in patients suspected of having BSI after cardiac surgery.

**Methods:**

Two hundred seventy-nine blood samples from 169 individuals with suspected BSI were analyzed by SF and BC. After excluding results attributable to contaminants, a comparison between the two groups were carried out. Receiver operating characteristic (ROC) curves were generated to determine the accuracy of clinical and laboratory values for the prediction of positive SF results.

**Results:**

14.7% (*n* = 41) of blood samples were positive using SF and 17.2% (*n* = 49) using BC (n.s. [*p* > 0.05]). In six samples SF detected more than one pathogen. Among the 47 microorganisms identified by SF, only 11 (23.4%) could be confirmed by BC. SF identified a higher number of Gram-negative bacteria than BC did (28 vs. 12, χ^2^ = 7.97, *p* = 0.005). The combination of BC and SF increased the number of detected microorganisms, including fungi, compared to BC alone (86 vs. 49, χ^2^ = 13.51, *p* < 0.001). C-reactive protein (CRP) (21.7 ± 11.41 vs. 16.0 ± 16.9 mg/dl, *p* = 0.009), procalcitonin (28.7 ± 70.9 vs. 11.5 ± 30.4 ng/dl, *p* = 0.015), and interleukin 6 (IL 6) (932.3 ± 1306.7 vs. 313.3 ± 686.6 pg/ml, *p* = 0.010) plasma concentrations were higher in patients with a positive SF result. Using ROC analysis, IL-6 (AUC 0.836) and CRP (AUC 0.804) showed the best predictive values for positive SF results.

**Conclusion:**

The SF test represent a valuable method for rapid etiologic diagnosis of BSI in patients after cardiothoracic surgery. In particular this method applies for individuals with suspected Gram-negative blood stream. Due to the low performance in detecting Gram-positive pathogens and the inability to determine antibiotic susceptibility, it should be used in addition to BC only (Pilarczyk K, et al., Intensive Care Med Exp ,3(Suppl. 1):A884, 2015).

## Background

Nosocomial infections represent the main non-cardiac complication after cardiovascular surgery and are associated with substantial morbidity, increased mortality, prolonged hospitalization and, eventually, economic burden [2, 3]. Respiratory tract infections account for more than half of all nosocomial infections after open heart surgery followed by surgical site infections and bloodstream infections (BSI) with a prevalence of approximately 20% [4]. Patients with BSI have a 4.2-fold increased risk of death, compared with non-infected patients [5]. Environmental contamination could be responsible of nosocomial infection acquisition and diffusions of multi drug resistance microorganisms (MDR) [6, 7]. Current guidelines highlight the importance of rapid administration of the most appropriate antimicrobial treatment to improve the survival of patients with suspected BSI and sepsis [8]. This is of special importance in respect of the problem of the ongoing antimicrobial resistance. The “gold standard” for the diagnosis of BSI is blood culture (BC) with pathogen identification and consecutive drug susceptibility testing. However, this process regularly requires at least 24 to 72 h. Sensitivity of BC is low due to uncultivable or fastidious microorganisms, polymicrobial or invasive fungal infections, or administration of anti-infectives prior to blood sampling [9]. Lee et al. reported that 73% of pathogens were detected with the first blood cultures, 90% with two, 98% with three, and 99.8% with four different consecutive blood cultures [10]. In addition, discrimination between infection and potential contamination is sometimes difficult. Thus, there is an urgent need to establish a rapid, sensitive, and specific method for detection of bacterial and fungal pathogens to improve management of patients with suspected BSI. PCR-based technologies have emerged over the last two decades and could represent an appropriate diagnostic tool in terms of sensitivity and speed of pathogen detection, in particular in life-threatening infections.

The LightCycler® SeptiFast (SF) is a multi-pathogen probe-based real-time PCR system targeting DNA sequences of 25 commonly observed bacteria and fungi present in blood samples within a few hours. However, data about the impact of PCR-based diagnostics on clinical decision-making process and modification of empirical antimicrobial therapy are very limited. A recently published prospective randomized trial demonstrated that in addition to a reduction in the time required for initial pathogen identification, the use of PCR was clearly able to reduce the time required for therapy modification from 38 to 19 h, however without reaching statistical significance [11]. Currently, there are no data about the accuracy and the impact of PCR based detection of BSI in patients undergoing cardiac surgery. Therefore, the aim of our study was to compare the performance of SF with conventional BC system in patients suspected of having BSI after cardiothoracic surgery.

## Methods

### Patients

In this retrospective observational study, data were collected between January 2009 and February 2013 on all consecutive patients with SF at our Intensive Care Unit (ICU), Department of Thoracic and Cardiovascular Surgery, West German Heart Centre Essen, Germany, in our institutional database. The diagnosis of the suspected BSI was made by clinical judgment by the treating physicians on basis of the occurrence of systemic inflammatory response syndrome (SIRS)/Sepsis criteria [8]. The decision of using SF was made either by the treating physicians or the infectious disease specialists. Data analysis was performed after the collection period. The study was approved by the Institutional Review Board according to the Declaration of Helsinki. All of the patients had previously granted permission for use of their medical records for research purposes. This written informed consent was obtained within the preoperative surgical written and verbal information conversation.

Patients were considered for inclusion in the study only if the met the following criteria:

(I) Suspected bacterial or fungal BSI
(II) Collection of paired blood samples for SF and at least two sets of BCs (two aerobic and two anaerobic bottles) from a peripheral vein or a central venous line at the same time point (within two hours)

The BC and SF results were compared separately by positivity of samples and by detected species of microorganisms/isolates.

### Blood cultures

Blood samples (at least two pairs of aerobic and anaerobic BC bottles, volume of 8–10 mL each) were collected by sterile venipuncture or from a central venous catheter (CVC) after disinfection of the connector and inserted into aerobic and anaerobic bottles and were sent to the laboratory. Samples then were incubated into the Bactec 9240 Plus (Becton Dickinson, Heidelberg, Germany), an automated microbial detection platform based on the colorimetric detection of CO_2_ produced by growing microorganisms. BC bottles were incubated up to seven days. In case of a positive signal on the Bactec instrument, 10 μL blood from aerobic blood culture was plated onto chocolate agar, blood agar, MacConkey agar, chromogenic yeast medium, and, if anaerobic bottle were positive, additionally onto two solid anaerobic media (Beerens and Schaedler agar; all from Oxoid, Wesel, Germany). Identification and susceptibility testing was performed according to the EUCAST (European Committee on Antimicrobial Susceptibility Testing) standard using the matrix-assisted laser desorption/ionization time-of-flight mass spectrometry VITEK MS, the VITEK2 (both bioMérieux, Nürtingen, Germany) and WalkAway MicroScan (Beckman Coulter, Krefeld, Germany) [12].

### SeptiFast

The LightCycler® SeptiFast test M Grade (Roche Molecular Systems, Mannheim, Germany) is an in vitro nucleic acid amplification test for the detection of bacterial as well as fungal DNA in human blood. It allows the identification of 25 bacterial and fungal species (see Table 1), being responsible for approximately 90% of all bloodstream infections. SF is the first real-time PCR-based system to be awarded a Conformité Européenne (CE) mark for pathogen detection and identification in suspected bloodstream infection. The analytical sensitivity of the assay, as indicated by the manufacturer, is between three and 100 colony forming units (CFU)/ml, depending on the microorganism. Following the manufacturer’s instructions, DNA was extracted and was amplified by the LightCycler® in three individual reactions (Gram-positive bacteria, Gram-negative bacteria, and fungi). To exclude false-negative results the test includes an internal control, provided by the SeptiFast kit. PCR products were simultaneously detected by fluorescence and melting temperature analysis, using specific hybridization probes and identification software.

**Table 1:** Analytical spectrum of the LightCycler® SeptiFast test

Gram-positive bacterial species	Gram-negative bacterial species	Fungal species
*Staphylococcus aureus*	*Escherichia coli*	*Candida albicans*
*Staphylococcus epidermidis*	*Klebsiella pneumoniae*	*Candida tropicalis*
*Staphylococcus haemolyticus*	*Klebsiella oxytoca*	*Candida parapsilosis*
*Streptococcus pneumoniae*	*Serratia marcescents*	*Candida krusei*
*Streptococcus pyogenes*	*Enterobacter cloacae, Enterobacter aerogenes*	*Candida glabrata*
*Streptococcus agalactiae*	*Proteus mirabilis*	*Aspergillus fumigatus*
*Streptococcus mitis*	*Pseudomonas aeruginosa*	
*Enterococcus faecium*	*Acinetobacter baumannii*	
*Enterococcus faecalis*	*Stenotrophomonas maltophilia*	

For coagulase-negative staphylococci and streptococci, a semiquantitative analytical cut-off value has been set by the manufacturer for distinguishing between true pathogens and contaminants from the skin flora

### Discrimination between infection and contamination in BC

Coagulase-negative staphylococci (CoNS), *Streptococcus* spp., *Corynebacterium* spp., or *Bacillus* spp. are frequent contaminants of BCs. To discriminate between true BSI and contamination, an algorithm based on a previous study was applied. A true BSI was considered if the patient has at least three SIRS criteria or two SIRS criteria and a CVC or other prosthetic material [13]. Positive findings for fungi were interpreted according to the European Organization for Research and Treatment of Cancer/Invasive Fungal Infections Cooperative Group and the National Institute of Allergy and Infectious Diseases Mycoses Study Group diagnostic classification of fungal infections [14].

### Discrimination between infection and contamination in SF

Isolates identified by PCR were considered to be pathogens or contaminants using a modified algorithm, combining microorganism pathogenicity, interpretation of blood culture results, and clinical, laboratory, and microbiological data [15]. The threshold of the SeptiFast software, based on the bacterial DNA amount, excluded CoNS and streptococci from the positive results and considered them contaminants. Fungal pathogens were categorized as described above.

### Statistical analysis

Statistical analyses were performed with SPSS Statistics 19 (IBM, Chicago, IL). Continuous data were expressed as median ± 95% confidence interval (CI); categorical data were expressed as percentage. Comparisons between two groups were carried out using unpaired Student’s t-test for normally or the Mann-Whitney Rank Sum Test for non-normally distributed data. Multiple groups were compared with ANOVA. Univariate analysis was performed on the quantitative variables using the Student t-test or Mann-Whitney test and on the qualitative variables using the Chi^2^ test of Fisher’s exact test.

To measure the sensitivity and specificity of laboratory and clinical data at different cut-off values, a conventional receiver operating characteristic (ROC) curve was generated. All variables showing a *p*-value of less than 0.1 between the two groups using Student t-test, Mann-Whitney test, Chi^2^ test of Fisher’s exact test were selected for ROC analyses. The optimal cut-off concentration was defined by the highest Jouden index (J = sensitivity + specificity – 1). Statistical significance was assumed for a *p*-value < 0.05.

## Results

During the study period, 279 matched blood samples from 169 patients suspected of having BSI were analyzed with conventional BC and SF. Out of these, 78% (132/169) were under antibiotic treatment at this time. Contaminants were significantly more frequent among blood cultures than SeptiFast (23 [8.2%] vs. 2 [0.71%], *p* < 0.001).

**Fig. 1:**
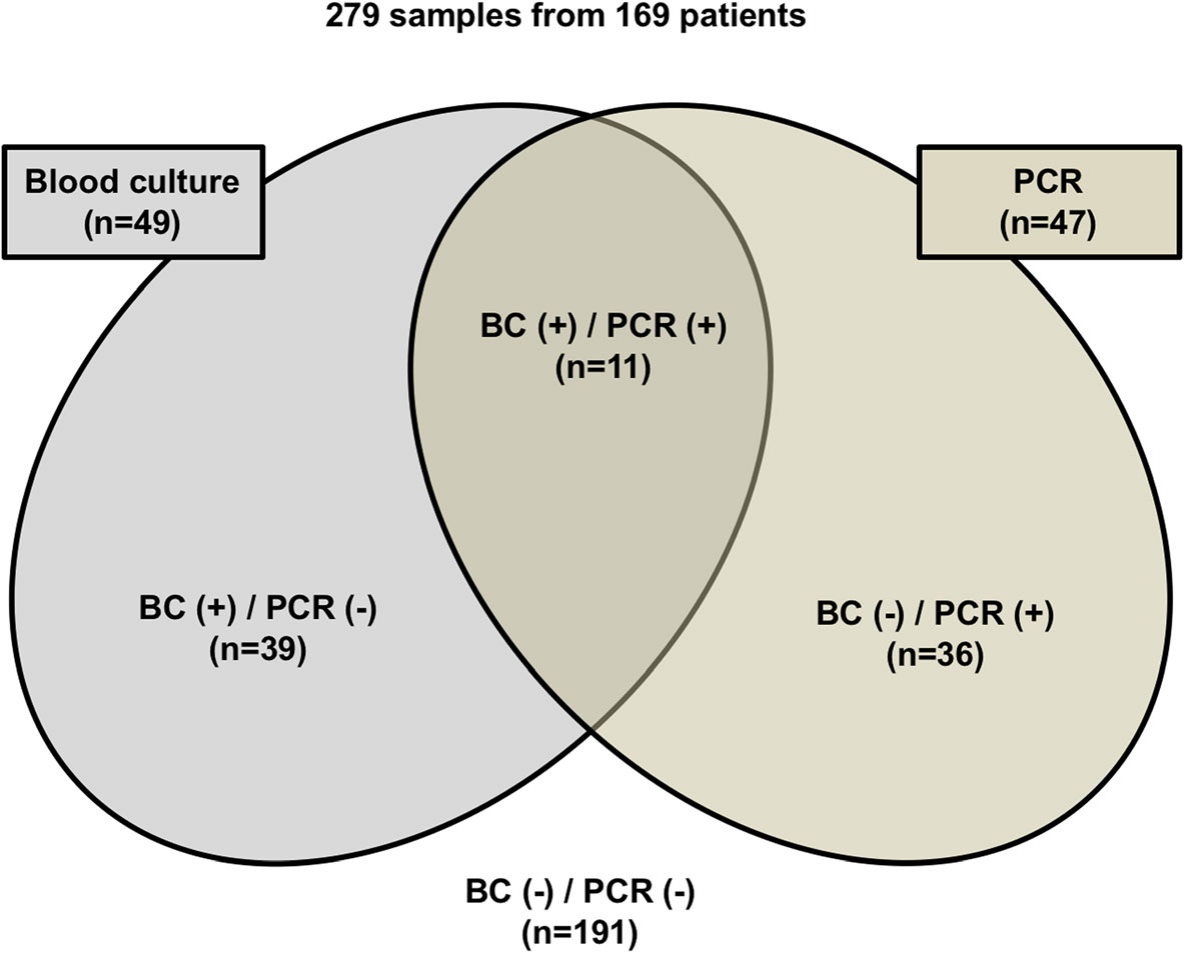
Number of detected microorganisms classified as infection in PCR, blood culture

After excluding contaminants, SF identified 47, while BC identified 49 episodes of BSI (χ^2^ = 0.05, *P* = 0.822). As illustrated in  Fig. 1, SF exclusively detected 36 pathogens that were missed by BC, whereas BC detected 39 pathogens in SF-negative individuals. Thus, 86 positive episodes of BSI were identified with the combination of both methods, being significantly higher than with SF or BC alone (χ^2^ = 10.86, *p* < 0.001 for SF and χ^2^ = 12.35, *p* < 0.001 for BC). BC analyses resulted in 51% sensitivity, 83% specificity, 46.7% positive predictive value (PPV), and 54.1% negative predictive value (NPV) whereas SF resulted in 51% sensitivity, 84% specificity, 46.4% PPV, and 54.4% NPV. s.

With BC, CoNS was the most frequently detected agent (13/49, 26.5%) followed by *E. faecium* (10/49, 20.4%) and *Candida* spp. (9/49, 18.4%). In contrast, the most frequently observed pathogen in SF was *Candida* spp. (9/47, 19.1%) followed by *Enterobacter* spp. (8/47) and *Klebsiella* spp. (7/49, 14.3%).

SF identified 7/33 (21%) Gram-positive bacteria, 28/35 (8%) Gram-negative, and 12/67 (67%) fungi, while BC identified 28/33 (85%), 12/35 (34%), and 9/18 (50%), respectively (Table 2).

Gram-negative detection rate was significantly higher with SF than with BC (χ^2^ = 14.93; *p* < 0.001), but for fungi, the difference to BC was not relevant (χ^2^ = 1.03; *p* = 0.3). In contrast, detection rate of Gram-positive bacteria was significantly higher with BC compared to SF (χ^2^ = 25,09; *p* < 0.001).

SF identified 36 pathogens that were not found in BC, while BC detected 39 pathogens in SF negative specimens. Microbial strains exclusively identified by SF were: *E. coli* (*n* = 3), *Klebsiella* spp. (*n* = 6), *E. faecium* (*n* = 4), *Enterobacter* spp. (*n* = 7), *E. faecalis* (*n* = 1), *P. aeruginosa* (*n* = 3), *A. fumigatus* (*n* = 3), *C.* spp. (*n* = 6), *S. marcescens* (*n* = 3), *S. maltophilia* (*n* = 1). BC detected the following pathogens in SF negative samples: CoNS (*n* = 13), *E. faecium* (*n* = 10), *C. albicans* (*n* = 6), *S. marcesens* (*n* = 3), *Klebsiella* spp. (*n* = 2), *S. aureus* (*n* = 1), *E. coli* (*n* = 1), other *Streptococcus* spp. (*n* = 1), *E. faecalis* (*n* = 1), M. morganii (*n* = 1). Polymicrobial infections were observed in seven patients. Five episodes were detected by SF; while BC identified multiple agents in only four specimens.

### Predictors for SF positivity

Several variables of the patients with and without pathogen identification in SF and BC were compared, respectively (Table 3). Whereas baseline demographics, gender, BMI, EuroScore-2 and SAPS and TISS on the day of admission on ICU as well as type of surgery did not differ between the two groups, patients with positive PCR were significantly younger than patients with negative PCR (57 years [51.7–68.0] vs. 68.0 [64.3–70.0], *p* = 0.01). In addition, prevalence of acute kidney Injury (AKI) with need for renal replacement therapy (RRT) was higher in SF positive patients (76% vs. 53%, *p* = 0.01).

**Table 2:** Detected microorganisms after exclusion of contaminations

	Number of isolates					
Pathogens	Total	Detected by PCR	Detected by BC	PCR pos/BC pos	PCR pos/BC neg	PCR neg/BC pos
Gram-positive bacteria	33	7 (21%)	28 (85%)	2 (6%)	5 (15%)	26 (79%)
*Staphylococcus aureus*	2	1 (50%)	2 (100%)	1 (50%)	0 (0%)	1 (50%)
*CoNS*	13	0 (0%)	13 (100%)	0 (0%)	0 (0%)	13 (100%)
*Streptococcus pneumoniae*	0	0	0	0	0	0
*Other Streptococcus spp.*	1	0 (0%)	1 (100%)	0 (0%)	0 (0%)	1 (100%)
*Enterococcus faecalis*	3	2 (67%)	2 (67%)	1 (33%)	1 (33%)	1 (33%)
*Enterococcus faecium*	14	4 (29%)	10 (71%)	0 (0%)	4 (29%)	10 (71%)
Gram-negative bacteria	35	28 (80%)	12 (34%)	6 (17%)	22 (63%)	7 (20%)
*Escherichia coli*	5	4 (80%)	2 (40%)	1 (20%)	3 (60%)	1 (20%)
*Pseudomonas aeruginosa*	5	5 (100%)	2 (40%)	2 (40%)	3 (60%)	0 (0%)
*Enterobacter cloacea*	8	8 (100%)	1 (13%)	1 (13%)	7 (88%)	0 (0%)
*Klebsiella pneumonia*	9	7 (78%)	3 (33%)	1 (11%)	6 (67%)	2 (22%)
*Stenotrophomonas maltophilia*	1	1 (100%)	0 (0%)	1 (100%)	0 (0%)	0 (0%)
*Corynebacterium spp*	0	–	0	–	–	0
*Morganella morganii*	1	–	1 (100%)	–	–	1 (100%)
*Serratia marcensens*	6	3 (50%)	3 (50%)	0 (0%)	3 (50%)	3 (50%)
Fungi	18	12 (67%)	9 (50%)	3 (17%)	9 (50%)	6 (17%)
*Aspergillus fumigatus*	3	3 (100%)	0 (0%)	0 (0%)	3 (100%)	0 (0%)
*Candida albicans*	15	9 (60%)	9 (60%)	3 (20%)	6 (40%)	6 (40%)

*BC* blood cultures, *CoNS* coagulase-negative staphylococci, *neg* negative, *PCR* polymerase chain reaction (SeptiFast assay), *pos* positive, *spp.* species

Laboratory markers of inflammation differed significantly between groups: C-reactive protein (CRP) (21.7 mg/dl ±11.41 vs. 16.0 ± 16.9, *p* = 0.009), procalcitonin (PCT) (6.6 ng/ml [2.7–16.4] vs. 3.1 [2.3–4.7], *p* = 0.015) as well as interleukin 6 (IL-6) (235.0 pg/ml [83.5–1582.2] vs. 72.3 [46.5–104.7], *p* = 0.010) were significantly higher in patients with positive SF result. In contrast, patients with negative PCR had a significantly higher WBC than patients with positive PCR (14.0 [13.0–15.0] vs. 12 [10.3–15.0], *p* = 0.014).

Patients with proven BSI in SF suffered from a more complicated postoperative course with prolonged ICU stay compared to SF-negative patients (ICU stay [days]: 26.1 ± 16.2 vs. 19.4 ± 12.8, *p* = 0.019). Comparing patients with positive and negative BC, demographics, inflammatory markers and organ function did not differ whereas ICU-stay was longer in individuals with positive blood culture (16 days [15–19] vs. 18.5 [14.0–26.2], *p* = 0.044).

**Fig. 2:**
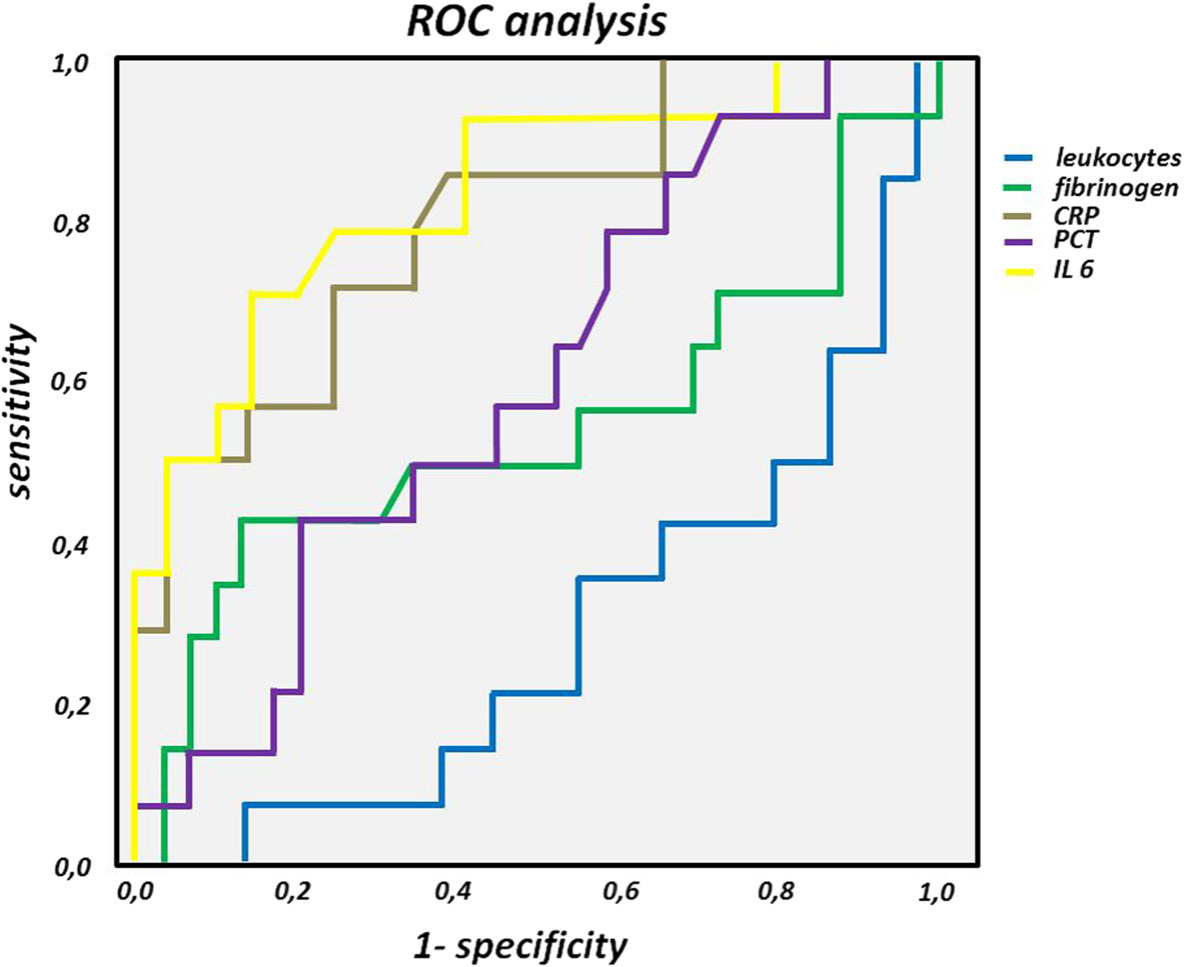
Receiver operator characteristic (ROC) curve for the prediction of SF positivity.

Using ROC analysis, IL-6 (AUC 0.836, sensitivity 78.6%, specificity 75.9% for a cut-off 184 pg/ml) as well as CRP (AUC 0.804, sensitivity 71.4%, specificity 75.9% for a cut-off 15.25 mg/dl) showed the best predictive values for positive SF results (Fig. 2). In contrast, PCT and leukocytes were associated with poor predictive capacity.

### Impact of SF on antimicrobial therapy

In eight out of 37 cases with pathogens solitarily identified by SF (21.6%) microbiological diagnostic information led to therapy adaptations (Table 4). Only one of these pathogens was detected by blood culture whereas the other seven remained undetected with conventional diagnostics. In three patients, detection of *A. fumigatus* in SF led to the addition of antifungal therapy with voriconazole, in another three patients therapy was escalated with fluconazole and caspofungin, respectively. In one patient, vancomycin was added due to *E. faecium* identification in SF. 50% of patients could be discharged home whereas four patients died during the further hospital course.

**Table 3:** Characteristics of patients with positive SF/BC result compared to those with negative SF/BC results

	PCR (SeptiFast)			Blood culture		
	Negative *(n = 237)*	Positive *(n = 42)*	*P*-value	Negative *(n = 232)*	Positive *(n = 47)*	*P*-value
Age [years]	68.0 [64.3–70.0]	57 [51.7–68.0]	**0.010**	67 [62.0–69.0]	69 [59–69]	n.s.
Male Gender [n, %]	161 (68)	30 (71)	n.s.	163 (70)	28 (59)	n.s.
Operative Procedure [n, %]						
CABG	47 (20)	9 (21)	n.s.	46 (20)	10 (21)	n.s.
Isolated AVR	8 (3)	1 (2)	n.s.	9 (4)	0 (0)	n.s.
Isolated MVS	5 (2)	1 (2)	n.s.	4 (2)	2 (4)	n.s.
Combined procedures	101 (43)	14 (33)	n.s.	99 (43)	16 (34)	n.s.
Aortic surgery	25 (11)	4 (10)	n.s.	24 (10)	5 (11)	n.s.
Thoracic transplant	13 (5)	4 (10)	n.s.	11 (5)	6 (13)	n.s.
Others	38 (16)	9 (21)	n.s.	37 (16)	8 (17)	n.s.
CPB time [min.]	177.0 [147.6–186.4]	149.0 [116.6–207.8]	n.s.	174.5 [147.2–185.0]	161.0 [139.7–198.9]	n.s.
Euro-Score II [%]	7.0 [5.2–10.1]	8.8 [2.9–21.0]	n.s.		10.5 [5.4–21.6]	n.s.
TISS-28 on day of SF/BC	19 [17–21]	21 [15–22]	n.s.	19 [17–21]	18 [14–21]	n.s.
SAPS on day of SF/BC	32 [31–34]	30 [27–35]	n.s.	32 [31–33]	31 [25–33]	n.s.
Oxygenation [mmHg/FiO_2_]	216.0 [196.3–230.4]	240.0 [210.2–269.5]	n.s.	220 [210.8–235.0]	214.5 [195.7–282.7]	n.s.
Heart frequency [min^−1^]	80.0 [80.0–90.0]	90.0 [83.4–106.6]	n.s.	90.0 [80.0–90.0]	90 [90.0–100.0]	n.s.
Body temperature [°C]	37.6 [37.4–37.8]	37.6 [37.1–37.9]	n.s.	37.6 [37.4–37.8]	37.6 [37.1–38.0]	n.s.
RRT [n, %]	125 (53)	31 (74)	**0.01**	129 (56)	27 (57)	n.s.
Laboratory values						
Serum lactate [mg/dl]	1.5 [1.4–1.8]	1.4 [1.1–1.9]	n.s.	1.5 [1.4–1.7]	1.4 [1.1–2.5]	n.s.
Bilirubin [mg/dl]	0.9 [0.7–1.0]	1.0 [0.8–1.6]	n.s.	0.9 [0.7–1.0]	0.9 [0.5–1.2]	n.s.
Leucocytes [/nl]	14.0 [13.0–15.0]	12 [10.3–15.0]	**0.014**	14.0 [13.0–14.0]	14.0 [10.5–16.0]	n.s.
Fibrinogen [mg/dl]	464.0 [430.0–496.7]	525.0 [389.9–594.2]	n.s.	472 [433.8–503.9]	504 [438.5–544.9]	n.s.
CRP [mg/dl]	14.4 [13.3–15.4]	14.5 [13.3–15.4]	**0.009**	14.8 [13.7–15.9]	15.2 [13.2–19.7]	n.s.
PCT [ng/ml]	3.1 [2.3–4.7]	6.6 [2.7–16.4]	**0.015**	3.4 [2.5–4.9]	2.2 [1.3–3.8]	n.s.
IL-6 [pg/ml]	72.3 [46.5–104.7]	235.0 [83.5–1582.2]	**0.010**	90.9 [61.7–144.3]	141.0 [46.6–240.2]	n.s.
ICU stay [days]	16 [15–19]	22 [16–33]	**0.019**	16 [15–19]	18.5 [14.0–26.2]	**0.044**
Hospital stay [days]	23 [19–29]	38 [25–59]	n.s.	27 [21–30]	28 [20.7–42.7]	n.s.

*AVR* Aortic valve replacement, *CABG* coronary artery bypass grafting, *CPB* Cardiopulmonary bypass, *CRP* C-reactive protein, *ICU* intensive care unit, *IL-6* interleukin 6, *LTX* lung transplant, *MVS* mitral valve surgery, *n.s.* not significant (*p* > 0.05), *PCT* procalcitonin, *POD* postoperative day, *RRT* renal replacement therapy, *SAP* Simplified Acute Physiology Score, *TISS* Therapeutic Intervention Scoring System, *TVS* tricuspid valve surgery

**Table 4:** Impact of SF on antimicrobial therapy

No.	Age range, gender	POD, Surgery	Identified pathogen in SF	Identified pathogen in BC	Initial antimicrobial therapy	Adjustment of therapy	Outcome after adjustment of therapy
*1*	50–59, 1	53, CABG, AVR, MVS	*Aspergillus fumigatus*	–	Meropenem, Vancomycin	Escalation with voriconazole	survived
*2*	50–59, 1	38, LTX	*Aspergillus fumigatus*	–	Meropenem, Vancomycin, Fluconazole	Escalation with voriconazole, discontinuation of fluconazole	died
*3*	70–79, 2	CABG, AVR, MVS, TVS	*Aspergillus fumigatus*	–	Piperacilline/Tazobactam	Escalation with voriconazole	died
*4*	50–59, 1	18, Aortic surgery	*Candida albicans*	*Candida albicans*	Ciprofloxacine, Vancomycin, Colistin	Escalation with caspofungin	died
*5*	60–69, 2	33, AVR, MVS	*Candida albicans*	*–*	Meropenem, Vancomycin	Escalation with fluconazole	survived
*6*	60–69, 1	47, AVR	*Candida albicans*	*–*	Linezolid, Imipenem	Escalation with caspofungin	survived
*7*	70–79, 1	21. CABG, MVS, TVS	*Enterococcus faecium*	*CoNS*	Piperacilline/Tazobactam	Escalation with vancomycin	died
*8*	20–29, 1	49. LTX	*Pseudomonas aeruginosa*	*–*	Vancomycin, Ceftazidime	Escalation with colistin	survived

*AVR* Aortic valve replacement, *BC* blood culture, *CABG* coronary artery bypass grafting, *CoNS* Coagulase-negative staphylococci, *LTX* lung transplant, *MVS* mitral valve surgery, *POD* postoperative day, *SF* SeptiFast, *TVS* tricuspid valve surgery. To ensure patient’s anonymity, gender was discriminated in 1 and 2

## Discussion

Our data demonstrate that the PCR-based SF test might represent a rational adjunct tool to the traditional BC method for rapid etiologic diagnosis of BSI in patients after cardiothoracic surgery. SF detects significantly more Gram-negative microorganisms than BC, whereas BC was superior regarding Gram-positive pathogens.

Early and reliable diagnosis of BSI and identification of bacteria and fungi is essential to initiate appropriate therapy in septic patients within one hour after sepsis as recommended by current guidelines [8, 16]. For decades, detection of pathogen microorganisms in patients with suspected BSI was mainly based on BC. However, this procedure per se has two intrinsic limitations: Firstly, this method is limited by the delay of 12–36 h for positive signaling and up to 72 for identification of the pathogen and the antimicrobial susceptibility profile. In addition, approximately 30% of pathogens remain undetected by BC and the time to positivity is longer for some fastidious bacteria, anaerobes, and fungi or under antimicrobial therapy [17]. Thus, there is an urgent need to improve the diagnostic tools for an improved management of patients with BSI or sepsis. Molecular methods, in particular the LightCycler SF, offer distinct advantages over blood cultures, including increased sensitivity and rapid diagnosis and is intensively investigated in clinical studies [9, 18]. However, diagnostic accuracy and cost–effectiveness should be established before implementation in clinical practice.

A meta-analysis including a total of 34 studies enrolling 6012 patients with suspected sepsis reported a high specificity with a modest and highly variable sensitivity [19]. Recent studies revealed a low sensitivity of the PCR method accompanied with a limited utility for the diagnosis of healthcare-associated BSI in critical care patients [20]. In contrast, another study including 104 critically ill patients suffering from SIRS showed that in 25 cases (16.9%, *n* = 148) rapid identification of involved pathogens by multiplex-PCR led to adjustment of therapy [21]. A randomized controlled trial enrolling 78 adults with suspected pulmonary or abdominal infection demonstrated a significant reduction in the time required for initial pathogen identification with SF compared with BC [10]. Even in the context of an increasing number of MDR rapid detection of the respective microorganisms is essential [22]. Taken together, the results about the usefulness of the SF for rapid detection of BSI in critical ill patients are divergent.

Patients after cardiothoracic surgery significantly differ from other cohorts: The use of cardiopulmonary bypass leads to a damage of the gastrointestinal mucosa, subsequent increased permeability, possible bacteremia, and the activation of a self-limited inflammatory response. The incidence of fungal infections especially in transplant recipients is, due to immunosuppression, higher than in the general ICU population. Commonly used biomarkers for bacterial infection might not work properly in the cardiothoracic population [16, 23].

In accordance with previous studies, the results of the present study demonstrate that SF, compared to BC, provided a better management of contaminants and a lower contamination rate [24]. In respect of CoNS interpretation and discrimination in BC clinical judgment must be used due to a lack of objective criteria. In contrast, in SF an automated software is used to identify contaminants, which explains the lower rate of contaminants.

In accordance with recently published data, we observed a clear superiority of SF in detecting Gram-negative organisms compared to conventional BC [25]. The reason for this discrepancy is unclear. Recent studies could demonstrate that the superiority of SF over BC is particularly observed in patients with severe sepsis [26]. In our cohort, patients with Gram negative BSI had higher concentration of CRP, IL-6 and PCT as well as a higher incidence of AKI with need for RRT compared to those with Gram-positive pathogens. Therefore, it might be hypothesized that SF is superior in detecting Gram-negative pathogens particularly in critically ill patients with severe infections.

BSI caused by Gram-negative bacteria is associated with a 7-fold increased risk of early mortality after cardiac surgery, compared with no BSI [5]. In contrast, BSI caused by Gram-positive bacteria other than *S. aureus* was only associated with a 2.2-fold increased risk of mortality [27]. Therefore, the early detection of Gram-negative bacteria in SF is of tremendous clinical relevance and might help to reduce mortality.

Since invasive fungal infections with Aspergillus are frequently associated with high morbidity and mortality, in particular immunocompromised patients benefit from prompt initiation of anti-fungal therapy [28]. However, the Surviving Sepsis Campaign does not recommend the routine use of empirical antifungals, based on the relatively low frequency of fungal causation of sepsis (∼5% of cases), although this is likely to rise. In our cohort of patients, a notably but not significant higher number of *Aspergillus* amplicons were detected by PCR as compared with BC. SF could improve patient outcome as a result of rapid and accurate fungi detection and the consecutive timely initiation of appropriate therapy [29]. Hence, one important clinical impact of SF seems to be the identification of otherwise undetected fungal BSI.

However, SF was inferior to BC in detecting Gram-positive bacteria including *S. aureus,* representing an important pathogen associated with high mortality. CoNS are a major constituent of human skin commensal flora, which were once considered relatively apathogen and a likely contaminant. But in patients with foreign materials (e.g. prosthetic valves, pacemakers, intravascular catheters) these organisms, due to their propensity to form a biofilm and to display resistance to multiple antibiotics, have increasingly been recognized as a cause of clinically significant infections. Thus, due to the significant number of infections that would be missed, SF could not replace blood culture for the identification of bloodstream infections. In addition, in SF pathogen identification is restricted to the 25 tested microorganisms and, moreover, susceptibility testing is not possible. In respect of the ongoing problem of multi drug resistance susceptibility testing is of increasing importance [30]. Therefore, SF cannot replace BC but represents an adjunct tool in combination with BC.

Even though in our study antimicrobial therapy was escalated due to the results of SF in eight patients, no de-escalation was done. As most of our patients were already on broad spectrum antibiotics and several blood cultures were drawn before choosing SF as diagnostic tool, empirical antibiotic therapy was considered to be adequate for most of the pathogens detected in SF and de-escalation was not done due to the lack of susceptibility testing.

Recent studies could demonstrate that use of new PCR based technologies in the management of septic patients lead to a significant reduction in treatment costs with a an average net saving of 9970 € per patient [31]. This economic benefit is mainly based on shortening of intensive care unit stay and the use of fewer antibiotics. However, the costs of SeptiFast (approximately 200–300 USD) are high compared to Blood Culture (approximately 30 USD).

Mencacci investigated the predictive role of procalcitonin in patients with suspected sepsis for positive test results in BC and PCR and revealed an area under the curve of 0.927 for SF positivity [32]. When applying a cut-off value of 0.37 ng/ml, the number of SF assays could be reduced by 53.9% with identifying 96.4% of pathogens. Leli et al. identified increased procalcitonin or white blood cells, fever > 38 °C, and low serum albumin as independent predictors of positive SF results in blood samples taken within 12 h after the onset of fever in 285 patients [33]. In our cohort, IL-6 as well as CRP was good predictors for SF positivity. Although PCT concentration are considered to be the gold standard of systemic inflammatory markers for diagnosis and evaluation of the treatment effectiveness, this marker only showed moderate predictive capabilities. The discrepancy could be due to the following: It is well established that aortic cross clamp and cardiopulmonary bypass related perioperative stress is associated with elevated PCT after cardiac surgery [34]. Several studies showed a poor correlation between elevated PCT concentration and bacterial infections or sepsis after major cardiac surgery [35]. Another aspect is that in our study 12 out of 47 positive SF results identified fungal pathogens. Thus, PCT as marker of bacterial infections is, anyway, not suitable for prediction of SF positivity in our cohort even more. Although the correlation of biomarkers and SF results are not very strong, in respect of the high costs of SF it might be helpful in the decision to perform SF or not.

## Limitations

There are several potential limitations to this study. First, our study suffers from the general limitation of a single-center, retrospective investigation: the results may not be applicable to other clinical settings with different patient characteristics, resources, and laboratory procedures. In addition, due to the small number of specific pathogens, the power to detect a difference between the groups is limited.

In interpreting the results of this study heterogeneity in the methods of drawing blood samples for BC must be considered as a limitation. It could not be ensured that all collected samples complied with the guidelines for drawing blood samples for BC, what can affect both for sensitivity and specificity [36].

A major limitation is the fact that there were no predefined criteria for performing PCR e.g. presence of more than two SIRS criteria. The algorithms used in this study to differentiate between contamination and infection of BC and SF were not evaluated in the cardiothoracic population. Therefore, the reliability of this algorithm in this setting is uncertain. However, as there is no published algorithm for cardiothoracic patients, we modified the originally published algorithm to incorporate specific characteristics of our patient’s cohort e.g. the presence of prosthetic heart valves or other extracorporeal devices.

Due to the retrospective nature of our study we could not ensure that the same blood sample was used for SF and BC.

It has to be mentioned that the SF test is not available in the United States yet.

## Conclusion

The PCR-based SF test might represent a valuable addition to the BC method for rapid etiologic diagnosis of bloodstream infections in patients after cardiothoracic surgery. This applies in particular for individuals with Gram-negative bacteremia. Since SF missed a certain number of Gram-positive pathogens, can only detect a limited number of pathogens and is unable to determine antibiotic susceptibility, it should always be used in conjunction with traditional blood culture methods.

## Abbreviations

AKI: Acute kidney injury
ANOVA: Analysis of variance
AUC: Area under the Curve
AVR: Aortic valve replacement
BC: Blood culture
BSI: Bloodstream infections
CABG: Coronary artery bypass grafting
CE: Conformité Européenne
CFU: Colony forming units
CI: Confidence interval
CoNS: Coagulase-negative staphylococci
CPB: Cardiopulmonary bypass
CRP: C-reactive protein
CVC: Central venous catheter
DNA: Deoxyribonucleic acid
ICU: Intensive Care unit
IL-6: Interleukin 6
LTX: Lung transplant
LVAD: Left ventricular assist device
MDR: Multi drug resistance microorganisms
MVS: Mitral valve surgery
NI: Nosocomial infections
NPV: Negative predictive value
PCR: Polymerase chain reaction
PCT: Procalcitonin
POD: Postoperative day
PPV: Positive predictive value
ROC: Receiver operating characteristic
RRT: Renal replacement therapy
SF: SeptiFast
SIRS: Systemic inflammatory response syndrome
spp.: species
TVS: Tricuspid valve surgery
